# Nanotherapeutics to Modulate the Compromised Micro-Environment for Lung Cancers and Chronic Obstructive Pulmonary Disease

**DOI:** 10.3389/fphar.2018.00759

**Published:** 2018-07-16

**Authors:** Dhruv R. Seshadri, Anand Ramamurthi

**Affiliations:** ^1^Department of Biomedical Engineering, Cleveland Clinic, Cleveland, OH, United States; ^2^Department of Biomedical Engineering, Case Western Reserve University, Cleveland, OH, United States

**Keywords:** non-small-cell lung cancer, chronic obstructive pulmonary disease, regenerative medicine, drug delivery, nanomedicine, tumor microenvironment, extracellular matrix, elastic matrix

## Abstract

The use of nanomaterials to modulate the tumor microenvironment has great potential to advance outcomes in patients with lung cancer. Nanomaterials can be used to prolong the delivery time of therapeutics enabling their specific targeting to tumors while minimizing and potentially eliminating cytotoxic effects. Using nanomaterials to deliver small-molecule inhibitors for oncogene targeted therapy and cancer immunotherapy while concurrently enabling regeneration of the extracellular matrix could enhance our therapeutic reach and improve outcomes for patients with non-small cell lung cancer (NSCLC) and chronic obstructive pulmonary disease (COPD). The objective of this review is to highlight the role nanomedicines play in improving and reversing adverse outcomes in the tumor microenvironment for advancing treatments for targeting both diseases.

## Introduction

Lung diseases are an increasing financial and physically taxing burden on the elderly population (Rijt et al., 2014). While our understanding of the pathophysiology of respiratory diseases have improved significantly over the past several decades due to advancements in imaging (instrumentation and image acquisition technology), therapeutic development (small molecules, monoclonal and bifunctional antibodies), and Big Data (deep learning, artificial intelligence), the diagnostic and clinical efficacy of currently available therapeutics to treat diseases such as non-small-cell lung cancer (NSCLC) and chronic obstructive pulmonary disease (COPD) remains extremely limited (Wang et al., 2017; Serra-Picamal et al., 2018). Approaches leveraging advancements in medicinal chemistry and materials science geared toward the site-specific repair of the compromised tissue causing these diseases could bridge the current gap that currently hinders treatment options for patients (Smola et al., 2008). Herein, we review some of the primary manifestations of NSCLCs and COPD as stand-alone or one occurring secondary to the other and discuss the use of intravenous or aerosolized nanotherapeutics to modulate the tumor microenvironment (TME) and regenerate and repair the lung extracellular matrix (ECM).

Lung cancer, the leading cause of cancer-related deaths worldwide, has a 5-year survival rate of ∼15% (Chen F. et al., 2007; Gridelli et al., 2009; Chen et al., 2014; Velcheti et al., 2014; Schalper et al., 2015; Mittal et al., 2016). The disease represents a tumor that originates from the respiratory epithelium. There are two primary types of lung cancer, namely, Small Cell Lung Cancers (SCLCs) and non-small-cell lung cancers (NSCLCs). NSCLC, which is the more prevalent of the two types, is sub-classified into multiple histologic subsets including adenocarcinomas, squamous cell carcinomas, and large-cell carcinomas (Chen F. et al., 2007). In the United States, ∼220,000 persons are diagnosed with lung cancer annually, of which more than 85% are NSCLCs (Chen et al., 2014). At the time of diagnosis, ∼50% of all NSCLCs are either localized or locally advanced and are subject to first line treatments that may involve resection (surgical removal of the tumor) or combined modality approaches (Chen et al., 2014).

For a majority of patients with NSCLC, systemic chemotherapy, oncogene targeted therapy, or immunotherapy are the primary modes of treatment (**Table 1**). A platinum based cytotoxic chemotherapy is commonly the first line treatment for advanced stage NSCLCs (stage IV). A chemotherapeutic is administered intravenously and systemically circulates to ultimately destroy cancerous tissue, but in the process, also detrimentally affects healthy tissues (Gupta et al., 2013). Dosing for such combination therapies is highly patient-specific and is determined by individual reactions to the chemotherapy combinations, which can be difficult to predict (Babu et al., 2013). Another limitation of these drugs is that they tend to be highly hydrophobic, which prevents them from being administered at high doses, since aqueous solubility is reduced. Clinical trials with biologic agents such has bevacizumab and cetuximab have shown minimal additional benefit when combined with cytotoxic chemotherapy and has resulted in excessive side effects (Goldstraw et al., 2011).

**Table 1:** Sampling of FDA approved therapies for NSCLC.

Company	Approved therapeutic
AstraZeneca	Gefitinib (Iressa): Kinase inhibitor blocks proteins that promote development of cancerous cells with certain EGFR mutations in exon 19 or 21 Osimertinib (Tagrisso): Kinase inhibitor for those with metastatic EGFR T790M mutation
Bristol-Myers Squibb	Nivolumab (Opdivo): Humanized IgG4 anti PD-1 monoclonal antibody which functions as a BRAF checkpoint inhibitor by blocking a signal that would have prevented T-cell activation
Eli-Lilly	Ramucirumab (Cyramza): Monoclonal antibody, Vascular Endothelial Growth Factor Receptor 2 (VEGFR2) inhibitor
Genentech	Bevacizumab (Avastin): Angiogenesis inhibitor via blocking of VEGF-A Atezolizumab (Tecentriq): Programmed death-ligand 1 (PD-L1) blocking antibody for patients with EGFR or ALK genomic tumor aberrations
Merck	Pembrolizumab (Keytruda): Therapeutic antibody that blocks the inhibitory ligand of PD-L1 receptors located on lymphocytes
Pfizer	Crizotinib (Xalkori): ALK and ROS-1 inhibitor

**FIGURE 1:**
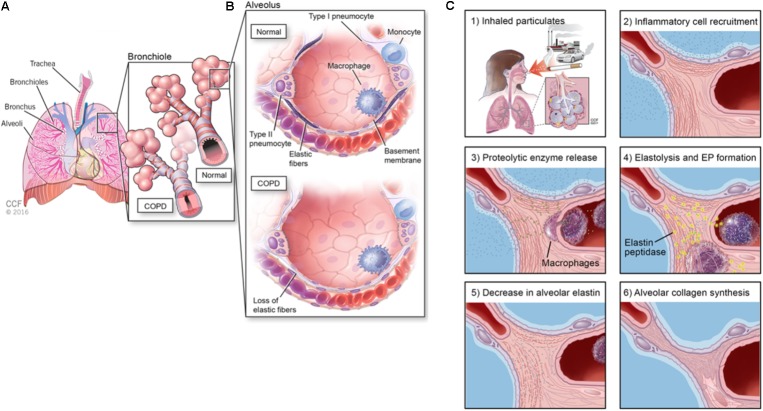
**(A)** Schematic showing anatomy of the lung, **(B)** alveolar changes in COPD and airway obstruction (<2 mm) diameter in the smaller conducting airways correlates with and is deemed a measure of COPD severity, and **(C)** etiology of alveolar elastic matrix breakdown following inhalation of particulates.

Despite the viability of first and second line treatments for NSCLC, there remains an unmet clinical need to also manage or reverse chronic obstructive pulmonary disease (COPD), which often occurs secondary to NSCLC (Houghton, 2013) and can compromise efficacy of treatments for NSCLCs. COPD remains a key risk factor for lung cancer and strongly correlates with chronic smoking, (**Figure 1**) (Houghton, 2013). Airway obstruction (<2 mm diameter) in the smaller conducting airways correlates with and is deemed a measure of COPD severity, (**Figure 1A**). There is strong evidence that physiologic changes deemed specific to COPD (e.g., breakdown of the elastic matrix into elastin peptides or EPs) promote tumor growth by polarizing macrophages from a pro-immunogenic, M1, to pro-tumorigenic, M2, phenotype (Curren Smith, 2015). Chronic bronchitis and emphysema are the two primary pathological manifestations of COPD (Houghton, 2013). Though airway fibrosis is not well characterized, it is assumed that constant inflammation in the small airway results in collagenous thickening of the walls of the small airways, (**Figure 1B**). In emphysema, the elastic structures of the gas exchange sacs (alveoli) are degraded resulting in their abnormal inflation and loss of gas exchange capacity, (**Figure 1C**) (Shifren and Mecham, 2006). Current treatments for COPD primarily target disease progression and are classified as pharmacologic (bronchodilators, corticosteroids), replacement (α1-antitrypsin modification), or supportive (oxygen delivery, pulmonary rehabilitation) (**Table 2**) (Ohnishi and Nagaya, 2008; Hind and Maden, 2011). Alternative strategies to not only limit, but also reverse COPD progression could thus be very valuable in terms of improving therapeutic prospects for patients receiving NSCLC therapies (Pandey et al., 2017).

**Table 2:** Sampling of COPD Drugs in development over the last 15 years (Yasothan and Kar, 2008; Mushtaq, 2014).

Company	Drug	Mechanism	Development phase
AstraZeneca	AZD-1236/9668/4818	Anti-inflammatory	Phase I
Bayer	BAY-71-9678	Elastase Inhibitor	Phase I
Boehringer Ingelheim	BEA-2180-BR	Anti-inflammatory	Phase II
GlaxoSmithKline/Theravance	Umeclidinium + vilanterol	Small molecule for targeting β_2_-AR + mAChR	Approved in United States Filed in EU
Medea	Midesteine	Elastase Inhibitor	Phase III
Novartis	Canakinumab	IL-1β antagonist	Phase I
Pfizer	Uk-432097	Adenosine-A_2a_ receptor agonist	Phase II

Successful simultaneous treatment of NSCLC and COPD rely on capabilities that are uniquely addressed by nanomaterials. Nanomaterials match the length scales of the inter-endothelial junctions in the blood vessels that feed tumors. This allows for the enhanced permeation and retention (EPR) of nanotherapeutics within tumor tissue (Chauhan and Jain, 2013; Chow and Ho, 2013). The EPR effect has shown to be the primary pharmacokinetic determinant for passive tumor targeting of cancer nanotherapeutics (Babu et al., 2013; Chauhan and Jain, 2013). Nanomedicines are advancing the field of immuno-oncology by their ability to deliver varied payloads with favorable molecular pharmacokinetics (Danhier et al., 2010). Additionally, the ability for nano-carriers to be conjugated with moieties (antibodies, ligands, peptides) that target biomolecules presented on the surface of cells, and their ability to provide sustained, steady-state, and predictable drug dosing can reduce potential cytotoxicity issues (Tong and Kohane, 2012; Tong and Langer, 2015). Since NSCLC- and COPD-afflicted tissues in the lung are in close proximity, there remains a need for targeted treatment modalities that would facilitate easy and non-systemic delivery of nanomedicines to provide tissue-localized and multi-functional therapeutic benefits.

In the cancer space, nanomaterials are used as carriers for (a) drug delivery, (b) contrast agents, and (c) for induced tissue ablation (Jain and Stylianopoulos, 2010; Bamrungsap et al., 2012). Such vehicles include, among others, liposomes, polymer-carriers, carbon structures (nanotubes, graphene), inorganic nanoparticles (NPs) (silica), and hybrid nanomaterials. The incorporation of chemotherapeutic agents within liposomal or biodegradable polymer (NPs) has improved drug solubility, slowed drug clearance, reduced drug resistance, and increased tumor filtration via the EPR effect. Metallic NPs (gold, iron oxide, silver) are effective in converting light to heat (e.g., IR radiation) via a photothermal effect to effectively destroy cancer cells. Recent innovations in developing inhalable therapeutics to target NSCLCs, when extended to NP delivery, have promise to circumvent the need for localized NP injections or systemic dosing strategies, thus making NP targeting to NSCLC straightforward and efficient. This article provides a comprehensive review of the use of nanomaterials to treat NSCLC and/or COPD with a dedicated focus on their use to modulate and repair the lung extracellular matrix.

## Overview of the Respiratory System

The respiratory system supplies oxygen to the body and is comprised of the diaphragm, chest muscles, nose, mouth, pharynx, trachea, bronchial tree, and lungs (**Figure 1A**). The bloodstream transports oxygen from the lungs to the rest of the body using hemoglobin as a carrier and returns carbon dioxide to the lungs to be exhaled. Inhaled oxygen passes through the pharynx enroute to the trachea, which divides into two main airways called the bronchi (Suki et al., 2011). The two lungs together comprise of five lobes, the right lung containing three and the left lung containing two. Within the lungs, the bronchi further divide into small bronchioles, which terminate in air sacs called alveoli. The oxygen transported into the lungs is finally transferred to the bloodstream via the walls of blood capillary within the alveoli. The alveoli are highly elastic and responsive to pressure changes from the air (Mecham, 1991; Shifren and Mecham, 2006). Following inhalation, the expansion of the lung causes the diaphragm to return to its smaller inter-breath size due to elastic recoil. The degree of stiffness of the lung tissue directly affects the air pressure needed to change lung volume (Mecham, 1991). A decrease in elasticity and increase in stiffness causes the lung to be less able to return to its normal size during exhalation.

## Extracellular Matrix of the Lung Tissue Microenvironment in NSCLC and COPD

**FIGURE 2:**
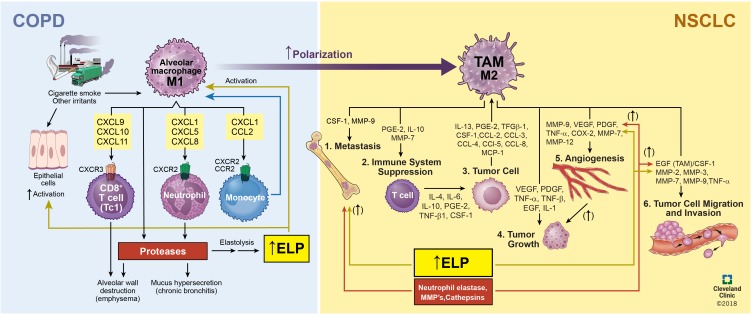
Relationship and interplay between COPD and NSCLC. Inhalation of inflammatory-generating particulates causes the degradation of the alveolar elastic matrix thereby decreasing O_2_, increasing sputum production, and prolonging exacerbations to bring about the onset of COPD. Continued worsening of COPD due to secretion of proteases, inflammatory neutrophils, angiogenesis formation due to vascular endothelial cells, and macrophage polarization from an M1, pro-inflammatory, to M2, pro-tumorigenic, phenotype brings about the onset of NSCLC.

The primary components of the lung ECM are glycosaminoglycans (GAGs), fibronectin fibrils, proteoglycans (PGs), laminin, heparan sulfate, nidogen/entactin, hyaluronate, chondroitin sulfate, matricellular proteins such as thrombospondin, tenascin X, and tenascin-C9, collagen (I, III, IV, V, and VI) fibers, and elastic fibers (Adjei and Blanka, 2015; Siegler et al., 2016; Yang and Gao, 2017). Collagen types I and III are the load-bearing fibers in the lung parenchyma while type IV assists in barrier function (Suki et al., 2011). In COPD (e.g., emphysema), the elastic fibers in the lung tissue are degraded due to enzymes upregulated by inflammatory cytokines and peptides whose generation is incited by more than 4,000 different inhaled particulates (**Figure 1**). Such inflammatory cascades can also promote tumor metastasis; thus, further understanding of the role of the TME in influencing pathogenesis of NSCLCs is imperative to improve efficacy of treatments for these cancers (**Figure 2**).

The TME has been shown to provide the necessary conditions for the growth and progression of metastatic cells (Gkretsi et al., 2015; Muntimadugu et al., 2017; Yang and Gao, 2017). Tumors contain both malignant and stromal cells that support the TME such as endothelial cells (ECs), smooth muscle cells (SMCs), fibroblasts, macrophages, stem cells, and vascular endothelial cells (VECs). Each of these cell types play a role in supporting the development and growth of tumors (Adjei and Blanka, 2015). Stromal cells are not inherently malignant and serve to maintain normal tissue structure and function (Li et al., 2007; Hoffman et al., 2011). Over time, they acquire a malignant phenotype that aid in metastasis through intracellular interactions or paracrine secretions by cancer cells. In this state, fibroblasts and immune cells generate chemokines and growth factors that galvanize cancer cell growth and recruit mesenchymal stem cells (MSCs) to replenish cells in the tumor (Hoffman et al., 2011). Additionally, the growth of the TME is known to degrade the ECM; thus, tissue engineering strategies are needed to modulate the TME and regenerate the ECM. The role of key stromal components (cells, ECM) surrounding the tumor, in promoting tumor growth are detailed below.

### Fibroblasts and Collagen

Fibroblasts are the dominant cell type in tumor stroma and produce different collagen subtypes, and fibronectin, which is a component of the tissue basement membrane (Driskell et al., 2013). Additionally, fibroblasts remodel the ECM through secretion of matrix metalloproteinases (MMPs) and other proteases (Driskell et al., 2013). The phenotypes of cancer-associated fibroblasts (CAFs) are different from healthy fibroblasts. The CAF express and secrete α-smooth muscle actin, platelet-derived growth factor (PDGF) which promotes cell proliferation and fibrosis, transforming growth factor beta (TGF-β), hepatocyte growth factor (HGF), insulin growth factor ½ (IGF), chemokines such as interleukin 1 (IL-1) and monocyte chemotactic protein 1 that facilitate the proliferation of cancer cells. The cells also secrete MMPs-2 and -9, and urokinase-type plasminogen activators (uPA), all of which degrade the elastic matrix in the lung parenchyma (Franco et al., 2010; Fullár et al., 2012).

### Elastic Matrix

The elastic matrix is responsible for the stretch and recoil property of lung tissue which withstands up to 200% strain (Shifren and Mecham, 2006). In the lung, the elastic tissue is of greatest abundance in the parenchyma (Mecham, 1991; Shifren and Mecham, 2006). The cells in the lung that are responsible for synthesis of elastin protein, the key component of elastic fibers, and for fiber neoassembly depend on anatomic location (Shifren and Mecham, 2006). For example, auricular chondroblasts produce the elastin found in the cartilaginous trachea and bronchi, pleura mesothelial cells deposit elastin in the pleura, and SMCs generate elastin in the lung vasculature (Shifren and Mecham, 2006). However, identifying the cell types responsible for elastic matrix within the lung parenchyma has been challenging since this area of the lung is composed of several different cell types and their respective phenotypes change during development. Elastic fiber formation is greatest in neonatal and fetal development (Mecham, 2008; Sivaraman et al., 2012). Myofibroblasts found near the capillary structures adjoining forming alveoli are considered the primary source of elastin at this site (Suki et al., 2011). The elastic matrix is particularly resilient and has a half-life of ∼80 years in humans (Balestrini and Niklason, 2015). Cigarette smoke extracts has shown to down regulate tropoelastin mRNA in rat fetal lung fibroblasts which Gao et al. (2005) attributed to the inhibition of transcription initiation and increased instability of lysyl oxidase (LOX; elastin crosslinking enzyme) mRNA transcripts (Gao et al., 2005). In an acellular model, cigarette smoke inhibited elastin cross-linking, while the transcription of LOX was reduced in fetal rat lung fibroblasts exposed to cigarette smoke extract (Gao et al., 2005; Plantier et al., 2007). Matrikines, which are products generated by degradation of the ECM, play key roles in stimulating progression of NSCLCs and in mediating inflammation and immune response (Maquart et al., 2004; Akthar et al., 2015). For example, both macrophages and neutrophils secrete elastase enzymes that degrade the elastic matrix. The generated elastin peptides recruit inflammatory cells to the lung (Plantier et al., 2007). These inflammatory cells generate potent reactive oxygen and reactive nitrogen species that lead to oxidative stress-related tissue damage (Almatroodi et al., 2014) (**Figure 1C**). Furthermore, these oxidant species adversely affect elastic fiber homeostasis by modulating the activity of proteinases (MMP-2, MMP-9, MMP-12), proteinase inhibitors, cross-linking enzymes (LOX), and activators (e.g., UPAs) rather than directly by impacting elastin (Almatroodi et al., 2014). Additionally, studies have shown that the pH of the ECM is also altered as a result of this degradation process (Tsuchiya et al., 2014; Balestrini and Niklason, 2015). One study showed that extremely basic pH (e.g., ≥10) during decellularization of lung tissue causes matrix damage. Since these tissues, when subsequently implanted *in vivo* stimulated an inflammatory response (Tsuchiya et al., 2014), it is assumed that the degraded ECM plays a major role in immune and inflammatory response. Adult cells are incapable of regenerating or repairing the elastic matrix as they poorly synthesize elastin precursors and are unable to polymerize and crosslink these precursors into functional, directionally oriented three-dimensional fibers; thus, there is a need to develop tissue engineering strategies to regenerate and repair the elastic matrix. Tangentially related, recent efforts in this author’s lab have been successful in augmenting elastic matrix neoassembly by aneurysmal SMCs utilizing doxycycline (DOX)-releasing polymeric NPs toward regressing abdominal aortic aneurysms (Sivaraman and Ramamurthi, 2013; Camardo et al., 2017). For example, the positive charge and hydrocarbon chains presented by cationic amphiphiles functionalized on these NPs simultaneously recapitulate the charge and steric hinderance-mediated anti-MMP effects of natural tissue inhibitors of MMPs (TIMPs) and also mimic the surface of low density lipoproteins (LDL), which electrostatically attract and augment activity of anionic LOX to increase elastic matrix assembly in tissues following their uptake (Jennewine et al., 2017; Sivaraman et al., 2017). Additionally, the long-term, steady-state delivery of DOX from these biodegradable NPs was shown to modulate inflammation by inhibiting MMPs and protecting α-1 antitrypsin, provide anti-oxidant effects, besides stimulating elastic matrix regenerative repair (Sivaraman and Ramamurthi, 2013; Camardo et al., 2017). Such approaches utilizing multifunctional NPs could be modified to aid in the regeneration and repair of the elastic matrix in the alveoli and inhibit macrophage polarization from a pro-inflammatory to pro-tumorigenic and pro-angiogenic phenotype (Hussain, 2016).

### Macrophages

Macrophages play a vital role in tumor progression in the lung. They are classified as M1 (classically activated) or M2 (alternatively activated) macrophages (Ngambenjawong et al., 2017). M1 macrophages are pro-inflammatory and control metastasis, suppress tumor growth, and induce a Th1 response whereas M2 macrophages induce a Th2 response and are pro-angiogenic and pro-tumorigenic (Martinez and Gordon, 2014). Each macrophage phenotype is characterized by a distinct profile of expressed cell surface marker proteins and ligands, and generated cytokines (Mosser and Edwards, 2008). Monocytes can be transformed into M0 macrophages in response to phorbol myristate acetate (PMA) and subsequently polarized to M1 macrophages in response to lipopolysaccharides (LPS) or interferon-γ (IFN-γ) (Kigerl et al., 2009). Studies have shown that toll-like receptor 4 (TLR4) and IFN-γ interact with the M1 activation signal (Mosser and Edwards, 2008; Martinez and Gordon, 2014). The major signaling pathways involved in transforming M1 macrophages are signal transducers and activators of transcription 1 (STAT1) and nuclear factor κB (NF-κB) (Kigerl et al., 2009). LPS stimulates TLR4 and activates NF-κB and interferon regulatory factor 3 (IRF3) which in turn promotes the secretion of pro-inflammatory cytokines that induce transformation of monocytes into the M1 phenotype (Mosser and Edwards, 2008). When activated to an M1 macrophage phenotype, these cells clear intracellular pathogens by releasing various pro-inflammatory cytokines to recruit and activate B and T cells during the early stages of inflammation (Mosser and Edwards, 2008). M1 macrophages are aggressively phagocytic and produce reactive oxygen species (ROS), nitric oxide (NO), high amounts of interleukins IL-12, IL-23, IL-18, IL-6, and low levels of IL-10, IL-1β, and TNF-α (Miao et al., 2017). M1 macrophages also express various membrane receptors for cluster differentiation proteins such as CD86, CD206, TLR4, and chemokine (C-C motif) ligand 5 (CCL5) and CCL2 (Miao et al., 2017). Post-inflammation, M0 or M1 macrophages are polarized by IL-4, IL-13, or IL-10 to transform into macrophages of M2a, M2b, or M2c anti-inflammatory states, respectively (Curren Smith, 2015). Among the three, M2a macrophages are the most studied and are herein generically referred to as M2 macrophages (Curren Smith, 2015). M2 signals are activated in response to IL-4R addition, wherein IL-4Ra, IL-4, and IL-13 activate STAT6 which causes the expression of M2 markers Arg-1, vascular endothelial growth factor (VEGF), high levels of IL-10, IL-12, IL-23, TGF-β, and IL-1Ra among other markers (Curren Smith, 2015). Additionally, the M2 phenotype expresses membrane receptor proteins such as CD163 (high affinity scavenger receptor for hemoglobin–haptoglobin complex), and CD206 (mannose receptor), and chemokines CCL17 and CCL22 (Curren Smith, 2015). Cigarette smoke contains nearly 4,000 toxins of which 55 are described as carcinogenic (Almatroodi et al., 2014). Nitrosamine 4 (methylnitrosoamino)-1-(3-pyridyl)-1-butanone (NNK), a nicotine derivative, has been shown to downregulate cytokine production of alveolar macrophages (AMs) and inhibit TNF and IL-12 while increasing IL-10 and PGD2 release thus modulating the phenotype of the AMs toward a pro-tumorigenic, pro-angiogenic state (Proulx et al., 2004).

Complete profiling of M1 and M2 phenotypic markers could greatly aid in developing combinatorial nanomedicine-based strategies to directly inhibit M1 to M2 macrophage polarization and also possibly repolarize them from an M2 back to the M1 phenotype. In this context, various drugs and nanomedicines have been successfully implemented to modulate phenotype of TAMs (**Figure 2**) and prevent macrophage polarization. Among such FDA-approved drugs are bisphosphonates (Pamidronate/Aredia; multiple myeloma, metastatic breast cancer), alkylating agents (trabectedin/yondelis; late-stage soft-tissue sarcoma), and tyrosine kinase inhibitors (Imatinib/Gleevec; chronic myologenous leukemia, CML) (Ngambenjawong et al., 2017). For example, the bisphosphonate (zoledronic acid) binds to microcalcifications in breast tumors and is phagocytosed by TAMs to induce apoptosis and repolarize M2 macrophages to the M1 phenotype (Ngambenjawong et al., 2017). Despite this, complete tumor regression has not been achieved by utilizing bisphosphonates. Bisphosphonates have been encapsulated for release from NPs or liposomes to improve the pharmacokinetics, reduce the toxic side effects, and alter biodistribution away from bone for extraskeletal applications (Rogers and Holen, 2011). Anti-angiogenesis therapies such as sorafenib and anti-VEGF antibody and liposomal doxorubicin (Doxil) have benefitted from the depletion of TAMs via bisphosphonates (Zeisberger et al., 2006). CD206 is one of the most commonly targeted receptors for macrophage delivery due to its overexpression on M2 macrophages (Zhu et al., 2013). The receptor can easily be conjugated onto nanomaterial surfaces but demonstrates low affinity (Ngambenjawong et al., 2017). To compensate for low binding, an anti-CD206 antibody has been developed for targeted TAMs; however, they are frequently sequestered in liver macrophages and liver sinusoidal ECs which also express high amounts of the same receptor (Irache et al., 2008). To circumvent such hurdles and improve TAM selectivity, researchers have designed nanocarriers utilizing the physical properties of the TME (Movahedi et al., 2012). Researchers fabricated mannosylated-polylactic-co-glycolide (PLGA) NPs masked with acid-sensitive PEG(2000) that prevents recognition by resident macrophages but is cleaved in the TME’s acidic environment thereby exposing the mannose receptor for targeted delivery to the TAM (Zhu et al., 2013). Superparamagnetic Iron Oxide NPs (SPIONS) have been utilized to repolarize M2 macrophages back to the M1 state by altering intracellular iron concentration. Recent studies have shown that ferumoxytol NPs could be useful to inhibit tumor growth by inducing M1 polarization to the M2 phenotype (Zanganeh et al., 2016). *In vitro*, the ferumoxytol NPs increased expression of genes associated with pro-inflammatory Th1 response in M1 macrophages which in turn caused an increase in caspase-3 activity in cocultured adenocarcinoma cells. *In vivo*, in female FVB/N mice, the NPs inhibited growth of subcutaneous adenocarcinomas and prevented hepatic metastasis (Zanganeh et al., 2016). It was also shown that an increased presence of M1 macrophages in the tumor tissue contributed to inhibiting tumor growth (Zanganeh et al., 2016). Glycocalyx-mimicking NPs have also been shown to repolarize M2 macrophages back to the M1 phenotype (Su et al., 2015). These glycol-NPs were self-assembled by the sugars galactopyranoside (Gal), mannopyranoside (Man), and fucopyranoside (Fuc) (Su et al., 2015). The Fuc sugar is instrumental in this phenotypic switch since it facilitates NP interaction with CD206 expressed by M2 macrophages (Su et al., 2015). In another study, polystyrene (PS) NPs surface conjugated with carboxyl (PS-COOH) and amino (PS-NH_2_) groups repolarized macrophages back to the M1 phenotype by reducing the expression of surface receptors CD200R and CD163 and by decreasing IL-10 levels (Fuchs et al., 2016). The underlying mechanism is poorly understood and needs to be further explored.

### Stem Cells

Cancer stem cells (CSCs) arise from stem cells present in healthy tissues. They are recruited from nearby tissues through circulation into the tumor stroma. CSCs arise from healthy somatic cells which develop oncogenic mutations that prevent them from entering at various stages of mitosis (Magee et al., 2012). Mesenchymal stem cells (MSCs) can be exploited for cancer immunotherapy and chemotherapy due to their ability to penetrate the lung tumor nodule when injected (Adjei and Blanka, 2015). Viral gene delivery methods have been utilized for secreting ligands from genetically engineered MSCs resulting in high transfection efficiencies (Loebinger et al., 2010; Shah, 2012). However, viral gene delivery poses numerous challenges such as marked immunogenicity which causes induction of the inflammatory system leading to degeneration of transduced tissue, toxin production, insertional mutagenesis, and limitation in transgenic capacity size (Loebinger et al., 2010; Shah, 2012). Non-viral gene delivery systems, wherein DNA is complexed with cationic polymers, such as polylysine or polyethylenimine (PEI), can circumvent hurdles posed with the delivery of viral vectors and have shown success in transfecting MSCs. Dendrimers with hydrophilic cores and hydrophobic coronas have delivered plasmid DNA to MSCs with limited cytotoxicity (Santos et al., 2010). Additionally, stem cells have been used as “Trojan horses” to deliver chemotherapeutics to tumors. MSCs encapsulated with drug-containing NPs migrate to tumors, where the NPs are released from MSCs by cell membrane rupture or stimulus-induced apoptosis of the MSCs (Levy et al., 2016). Levy et al. (2016) loaded MSCs with PLGA microparticles (MPs) encapsulated with the macromolecule G114, a thapsigargin-based prostate specific antigen (PSA)-activated prodrug (Levy et al., 2016). G114 released from G114 MP-loaded MSCs selectively induced the death of the PSA-secreting PCa cell line, LNCaP, and inhibited tumor growth when added to CWR22 PCa xenografts (Levy et al., 2016). NPs can also be conjugated to the cell membrane of MSCs to ensure cell survival toward subsequent tumor inhibition (Li et al., 2011). SMCs have aided in ECM regeneration and can be a worthwhile delivery vehicle to target the alveoli to regenerate the elastic matrix (Swaminathan et al., 2016). Our lab has recently demonstrated significant elastogenesis by bone marrow MSC-derived SMC-like cells (BM-SMCs) and their pro-elastogenic and anti-proteolytic effects on rat aneurysmal SMCs. The BM-SMCs were loaded with super paramagnetic iron oxide nanoparticles (SPIONs) toward guiding them to the AAA wall using an applied external magnetic field (Swaminathan et al., 2016). *In vitro*, the BM-SMCs stimulated elastin regeneration and attenuated proteolytic activity by cocultured aneurysmal SMCs both upon and without SPION labeling. This study showed that SPION-labeling of the BM-SMCs, to the extent necessary to impart the cells magnetic mobility in an applied magnetic field for uptake into matrix compromised vessels *in vivo*, are not cytotoxic and do not alter cell phenotype and ECM generation properties. In a study by Hoffman et al. (2011) the phenotype, clonogenicity, and differentiation potential of lung MSCs (L-MSCs) compared to BM-MSCs was studied to evaluate their *in vivo* survival, retention, paracrine gene expression, and repair of elastase injured tissue structures post-transplantation (Hoffman et al., 2011). Compared to BM-MSCs, L-MSCs showed greater survival, demonstrated higher number of CD45neg L-MSCs, and expressed higher levels of several transcripts (e.g., Ccl2, Cxcl2, Cxcl10, IL-6, IL-11, Hgf, and Igf2) *in vitro* (Hoffman et al., 2011). The study showed that both L-MSCs and BM-MSCs reduced elastase injury to the same extent and that tissue specific L-MSCs possess mechanisms that prolong retention in the lung after intravenous transplantation and produce greater healing of elastase injury comparable to BM-MSCs. This could potentially aid in maintaining alveolar homeostasis for emphysema (Hoffman et al., 2011).

### Vascular Endothelial Cells

Vascular endothelial cells line the lumen of blood vessels and facilitate the transport of nutrients and oxygen that support solid tumor growth. Tumor VECs differ from normal ECs in terms of their irregular shape, high motility, increased fenestration (prohibits small molecule therapeutics from reaching the TME), and ability to form leaky blood vessels (enables cancer cells to initiate metastasis) (Aird, 2012; Yamasaki et al., 2012). Cancer therapies have been utilized to target VECs with the goal of cutting off blood circulation to the tumor thereby “starving” the tumor of its necessary nutrients (Carmeliet and Jain, 2011; Fang et al., 2011). Since blood vessels are easier to target than cancer cells, targeted drug delivery to the tumor blood vessels would allow a higher drug concentration to reach and be retained in the tumor (Fang et al., 2011). VECs express integrins, proteoglycans, and proteases that can selectively bind to ligands conjugated on drug-laden nanocarriers. This can improve targeting of nanotherapeutics for more effective tumor inhibition (Desgrosellier and Cheresh, 2010). For example, aptamers such as nucleolin-conjugated to drug-laden NPs have shown to more effectively inhibit tumor growth than that of unmodified drug-NPs (Guo et al., 2011). Guo et al. (2011) conjugated AS1411 (Ap; a DNA aptamer which specifically binds to nucleolin and is highly expressed in the plasma membrane of both cancer cells and VECs) onto PEG-PLGA NPs for anti-glioma delivery of paclitaxel (PTX) (Guo et al., 2011). The Ap-nucleolin binding prolonged time spent by the NPs in circulation and enhanced association of the NPs with C6 glioma cells and subsequent accumulation of the released PTX in the tumor. Thus, tumors treated with the AP-nucleolin-conjugated PTX-NPs more effectively inhibited tumors in mice bearing C6 glioma xenografts and rats bearing intracranial C6 gliomas relatively non-surface modified PTX-NPs and exogenous delivery of Taxol^®^, which served as the control. Other useful targeting moieties include VEGFR-1, VEGFR-2, and α_5_β_3_ all of which are overexpressed by ECs of leaky blood vessels feeding the tumor (Brannon-Peppas and Blanchette, 2004). The K237-(HTMYYHHYQHHL) ligand, a peptide which binds to VEGFR2 (KDR) receptors expressed on the surface of VECs to inhibit the VEGF-KDR angiogenic signal pathway, was conjugated to PTX-encapsulated aldehyde poly(ethylene glycol)–poly(lactide), PEG-PLGA NPs (Yu et al., 2010). The peptide-conjugated NPs were internalized by human umbilical vein endothelial cells (HUVEC) following K237-KDR engagement. This led to the improved anti-angiogenic activity deduced from mitigated HUVEC proliferation, migration, and tube formation. The K237-PTX-NPs demonstrated accurate *in vivo* tumor neovasculature targeting, long-term effects in terms of apoptosis of ECs of the tumor neovasculature, and necrosis of breast tumor tissues, when implanted in female BLAB/c nude mice. Such strategies can be modified for NSCLC to potentially arrest tumor growth and aid in modulating the ECM or TME.

## Nanomaterials to Modulate the Lung Tissue Microenvironment

Thus far, an emphasis has been placed on discussing various components of the lung TME and how tumor modulation could offer viable treatment platforms for lung cancer. In the following section, we highlight classes of prominent nanomaterial subtypes utilized in the context of NSCLC and COPD treatment. It is to the authors’ understanding that there remains an unmet medical need for the development of a combinatorial treatment for both diseases utilizing nanomaterials. Thus, this section reviews the application of nanomaterials for NSCLC and COPD individually.

### Non-Small-Cell Lung Cancers

#### Polymeric Nanomaterials

Biodegradable and synthetic polymers such as poly(lactic-co-glycolic) acid (PLGA), poly(lactic acid) (PLA), albumin, gelatin, polycaprolactone (PCL), polyethylene glycol (PEG), chitosan, alginate, and collagen have been utilized due to their release properties, size, and biocompatibility. Polymeric NPs have been extensively researched to deliver targeted chemotherapeutics to lung tumors and have been shown to enhance the efficacy of anticancer agents. PEG-Poly-L-Lactic Acid (PEG-PLLA) NPs encapsulated with taxanes demonstrated improved efficacy of chemoradiation therapy *in vitro* and in an A549 lung tumor xenograft model (Jung et al., 2012). A cremophor-free nanoformulation (Genexol-PM) has also been developed for treating lung cancer (Kim et al., 2007). This preparation contains paclitaxel and cisplatin encapsulated within NPs formulated using a block copolymer of PEG and PLLA and was tested in phase II clinical trials in patients with advanced NSCLC. Folic acid (FA)-conjugated PEG-Poly-L-Glycolic Acid (PEG-PLGA) NPs were fabricated for co-delivery of the chemotherapeutic agents cisplatin and paclitaxel (He Z. et al., 2016). The anti-tumor effects of these NPs were evaluated in blood compatibility assays and complement activation tests. The FA-PEG-PLGA NPs did not induce blood hemolysis, blood clotting, or complement activation and did not have cytotoxic effects. The co-delivery of the two chemotherapeutic agents suppressed growth of lung tumor xenografts and prolonged survival time of the xenografted mice. Another group developed a chitosan-based NP encapsulated with the anti-neoplastic agent lomustine (Mehrotra et al., 2011, p. 132). The efficacy of these NPs was demonstrated via its effects on L132 lung cancer cell line *in vivo* culture. Tseng et al. (2007) successfully delivered gelatin NPs containing epithelial growth factor (EGF)-targeted biotinylated (bEGF) that enhanced cellular uptake in EGFR overexpressing cancer cell lines. Based on the promising outcomes, this group further developed an aerosol of cisplatin-encapsulated gelatin NPs which was tested in a mouse model for lung cancer (Tseng et al., 2008). This mode of delivery lead to high cisplatin concentrations within the lung tumors, resulting in high anti-tumor activity.

#### Inorganic Nanomaterials

Inorganic NPs including noble metals (typically gold or silver) have been extensively studied and utilized for diagnostic sensing and imaging of NSCLCs, and as delivery vehicles to treat these conditions (Xiong et al., 2013). Gold-based particles (AuNPs) show particular promise, as they are able to offer multimodal theranostic approaches in a single formulation, serve as drug delivery agents, and serve as radiation enhancers (Cryer et al., 2015). One of the advantages of AuNPs is their ability to be surface-modified in a relatively straightforward manner by capitalizing on the strong interaction between gold and thiolate groups. Another benefit of AuNPs is that they possess significant photo-thermal properties. This can be a useful attribute in ablating cancer cells, and thus treating NSCLCs, with or without delivering anticancer drugs. In this context, AuNPs were utilized in photodynamic therapy (PDT) for the delivery of hydrophilic PDT agent purpurin-18-*N*-methyl-D-glucamine (Pu-18-NMGA) to A549 lung cancer cells (Chen Y.-H. et al., 2007). This approach resulted in a higher PDT activity than free Pu-18-NMGA thus suggesting its promise to ablate NSCLCs *in vivo*. AuNPs have also been used to physically deliver anticancer drugs to provide additional therapeutic benefit (Chen Y.-H. et al., 2007). Methotrexate (MTX), a chemotherapeutic and immunosuppressant used to treat a variety of cancers and rheumatoid arthritis, is hydrophilic, and has poor tumor retention times (Chen Y.-H. et al., 2007). To improve its retention within tumors and thus its therapeutic efficacy, MTX was conjugated to AuNPs, and these NPs were delivered intravenously in a Lewis Lung carcinoma lung model. AuNPs have also been utilized for gene delivery (Chen Y.-H. et al., 2007). PEG-modified gold NPs have been formulated, conjugated with RGD peptides and c-myc siRNA on the surface, and administered via an intra-tracheal instillation (Conde et al., 2013; Lee et al., 2016). These RGD-NPs enabled down-regulation of c-myc oncogene and tumor growth inhibition in mice containing CMT/167 mouse lung carcinomas (Conde et al., 2013). Silver NPs (AgNPs) are promising as nanocarriers due to silver exhibiting high conductivity, chemical stability, low toxicity, high therapeutic efficacy (antifungal, anti-inflammatory, antimicrobial, anti-angiogenesis, antiplatelet, and anticancer activities) (He Y. et al., 2016). AgNPs are non-toxic to the human body at low doses. AgNPs have been fabricated by physical, chemical, and biological methods. Green synthesis, utilizing plants or microorganisms, is emerging as an opportune method to synthesize AgNPs due to its ease of fabrication, efficiency, and “eco-friendly” nature (He Y. et al., 2016). Plant extracts have shown to be powerful reducing agents due to the presence of phenolic compounds demonstrating high antioxidant activity. Recently, the therapeutic effects of AgNPs on the lung cancer H1299 cell line was explored *in vitro* (Gengan et al., 2013). The work showed that AgNPs inhibit the growth of the H1299 NSCLC cells. Additionally, the AgNPs suppressed the growth of H1299 tumors when injected into a mouse H1299 xenograft tumor model (Gengan et al., 2013).

Hydroxyapatite (HAp) NPs have been utilized for the delivery of proteins and various biologics. Prior literature suggests that hydroxyapatite NPs (HApNPs) induce apoptosis in several cancer cell lines via a mitochondria-dependent pathway, oxidative stress, or inhibited protein synthesis resulting from aggregation of HApNPs within cancer cells (Sun et al., 2016). HApNP-induced cytotoxicity was compared between A549 NSCLC cancer cells and healthy bronchial epithelial cells (16HBE) (Sun et al., 2016). HApNPs induced a cytotoxic response in A549 cells but did not affect the viability of the 16HBE cells. These results show that HApNPs may be effective therapeutic agents for targeted treatment for NSCLC. HAp can also be doped with different metal ions such as iron, cobalt, nickel, or silver to enable their additional use as contrast agents for MRI and theranostics. Hafnium-doped HApNPs (Hf-HApNPs) have been used to enhance ROS for more effective anti-cancer treatment (Chen et al., 2014). A549 cells were utilized as the *in vitro* models to monitor the impact of ionizing radiation on these Hf-HApNPs (Chen et al., 2014). When the Hf-HApNPs were exposed to gamma-rays, the Hf-HApNPs generated increased ROS levels in cells to cause toxicity. This approach has promise to serve as a first or second-line treatment for NSCLC (Chen et al., 2014).

#### Dendrimers

Dendrimers are a class of macromolecular hyper-branched polymers exhibiting a well-defined, radial branching architecture. They have been tested as drug carriers, excipients, and even as active therapeutic agents (Kesharwani et al., 2014). The small size, spherical shape, and lipophilicity of dendrimers render them more effective as drug delivery vehicles relative to linear polymers due to their ability to penetrate cell membranes and increase conjugation efficiencies when targeted delivery to the TME is desired. Synthetic dendrimers can attach to hormones, antibodies, or liposomes. Their loading depends on dendrimer carrier properties, such as the number and type of active sites, external functional groups, loading space, and lipophilicity. siRNA delivered in tandem with chemically modified dendrimers have been shown to have high avidity for Tie2-positive ECs in the lung (Khan et al., 2015). These formulations have been shown to be useful to treat diseases resulting from or involving a dysfunctional endothelium and could thus be useful to inhibit cancer inflammation or metastasis. In another study, PEGylated polylysine dendrimers were used as vehicles for pulmonary drug delivery (Ryan et al., 2013). This study suggested that small PEGylated dendrimers may be delivered effectively to the bloodstream via the inhalation route and that larger sized PEGylated dendrimers may be retained in the lung for long term, controlled release. Further work is needed to translate these preliminary results to clinical trials for NSCLC treatment. Furthermore, a non-viral aerosol formulation of p53 gene (p53sm) and polylysine/protamine combination (AND) exhibiting low toxicity was recently developed to treat early lung cancers and bronchoalveolar carcinomas (Zou et al., 2007). This approach showed that AND is 3–17-fold more effective than commonly utilized cationic polymers (i.e., polyethyleneimine, PEI) to transfect NSCLC cells and the aerodynamic size (0.2–3 μm) of AND-p53sm is optimal for deposition in the human respiratory tract. Aerosolized AND-p53sm increased the lifespan of mice bearing orthotopic NSCLC xenografts compared to optimal Cisplatin administration. Additionally, NPs conjugated with ligands and antibodies for targeted delivery have been delivered via an aerosol to bind with specific receptors on the surface of cancer cells (Zarogouldis et al., 2012). Anticancer drugs, such as paclitaxel, cisplatin, doxorubicin, gemcitabine, camptothecin, azacytidine, and fluorouracil, which have been used to treat both SCLCs and NSCLCs, have been delivered via inhalation (Kuzmov and Minko, 2015).

Delivery of therapeutics via aerosols has gained prominence for COPD treatment as is described next.

### Chronic Obstructive Pulmonary Disease

Chronic obstructive pulmonary disease is characterized by mucus hypersecretion and severe inflammation of the chronic obstructive airway and degradation of the elastic tissue. Thus, the main form of passive-based treatments involves the use of anti-inflammatory drugs (e.g., corticosteroids and antibiotics) delivered via an aerosol for deposition within the airway or specified region of interest in the lung. Aerosol-based delivery is advantageous where rapid (seconds or minutes) systemic biodistribution of small-molecule therapeutics is required (Patton and Byron, 2007). Small molecules are rapidly absorbed into systemic circulation due to the large surface area of the lung, highly dispersed nature of the aerosol, good epithelial permeability, and small aqueous volume at the absorptive surface (Patton, 1996). Aerosol-based delivery represents the fastest rate of uptake of any delivery method apart from intravenous delivery. Additionally, inhaled drugs that enter circulation are less likely to be degraded than if they are delivered orally (Patton and Byron, 2007). Aerosol particles with an aerodynamic diameter of about 1–2 μm, when slowly inhaled, can be deposited in the lung with 90% efficiency, with the majority deposited in the peripheral airways that are rich in alveoli (Byron, 1986; Patton and Byron, 2007). Since the alveoli tend to contain macrophages, it is necessary and beneficial to formulate the drugs as liquids or as highly water soluble particles that dissolve rapidly in the lungs to avoid degradation by macrophages (Patton, 1996; Patton and Byron, 2007). Optimizing particle size is critical as too small of a particle could risk exhalation and particles that are too large tend to be deposited in the upper airways, mouth, and throat (Patton and Byron, 2007). As previously described, asthma and COPD are the most common target applications for inhalable therapeutics. Beclovent and Albuterol are therapeutics commonly used to treat asthma. Beclomethasone (or Beclovent) is a corticosteroid which prevents certain cells in the lung from releasing substances that cause asthma symptoms (Beclovent Advanced Patient Information, 2018). Albuterol is a bronchodilator that relaxes muscles in the airways and increases airflow to the lungs and is used to treat or prevent bronchospasms in people with COPD. Additionally, various types of β agonists, anticholinergics, corticosteroids, and anti-inflammatory drugs are delivered via inhalation (Sharma et al., 2001).

There are four classes of clinically successful aerosol devices which include dry powder inhalers (DPIs), nebulizers, soft-mist inhalers, and pressurized metered dose inhalers (pMDIs) (Lee et al., 2015). DPIs have been developed to overcome poor actuation-inhalation coordination during treatment of asthma and COPD. Several advantages of this device include high stability, non-invasiveness, delivery of hydrophobic drugs, storage at room temperature, and desirable drug release properties. DPI formulations consist of either a respirable active drug or drugs blended with a non-respirable excipient such as lactose, which is currently the only FDA approved non-respirable carrier (Ibrahim et al., 2015). Unlike DPIs, nebulizers and pMDIs are liquid-based aerosol systems. Nebulizers have been used in the clinic for many years and can treat elderly patients and young children (less than 2 years). Nebulized drug formulations exist as a suspension or solution and are then atomized into droplets. These devices are used when patients are unable to control their breathing or when they are receiving mechanical ventilation. Nebulizers present a viable approach to treat lung cancer via the delivery of chemotherapeutic drugs, especially those encapsulated within NPs, which allow sustained release of the drug over extended periods of time (Verma et al., 2013; Bianchi et al., 2014). An ideal nebulizer should maintain drug stability and not cause any changes in the drug formulation (White et al., 2005). Parameters known to affect the delivery efficiency of nebulized solutions include the solution viscosity and pH, surface tension, osmolarity, and drug or NP concentration. Device parameters that influence these parameters include aerosol flow rate and nebulization rate. Soft-mist inhalers utilize generated mechanical energy from the spring to expel the drug. It has been shown that aerosol velocity generated from this method traveled slower and had a longer duration than aerosols generated from a pMDI (Anderson, 2006). While nebulizers and soft-mist inhalers do not require specialized inhaled coordination, pMDIs need propellants such as hydrofluoroalkanes (HFAs) and are released via an orifice over short durations (Anderson, 2006). It has been shown that only 10–15% of the emitted aerosol can reach the lung parenchyma due to the lack of hand-mouth coordination and inspiratory flow rate (Anderson, 2006).

The aerosolized delivery of drug-laden nanomaterials for the site-specific and targeted repair of compromised tissue in the lung microenvironment is an emerging application for the respiratory field (Rijt et al., 2014). A major hurdle hindering the utility of NPs for COPD treatment is the inability to penetrate and compensate for the viscosity of the mucus layer. Researchers sought to investigate if N-acetyl cysteine (NAC) CF sputum treatment in combination with low-molecular weight PEG coatings on fluorescent carboxyl-modified PS nanoparticles (PS-COOH, 200 and 500 nm), can synergistically enhance particle penetration across fresh undiluted CF sputum (Suki et al., 2011). Research showed that N-acetyl cysteine treatment of CF sputum attributed to an increase in mesh spacing thereby permitting large fractions of the PEG-modified, non-mucoadhesive NPs to rapidly penetrate NAC-treated CF sputum (Suki et al., 2011). Such work has significant therapeutic implications for COPD where excess mucus accumulation and prolonged exacerbations occur. Recent research has also shown that the hydrodynamic diameter plays a crucial role for the mucociliary clearance of particles in CF patients (Rytting et al., 2008). Rytting et al. (2008) showed that small diameter PS particles (120 nm) moved more efficiently through the sputum of CF patients while larger diameter particles (270–560 nm) demonstrated significantly less mobility. While this study was not tested in COPD patients, it can be hypothesized that the particles could travel as efficiently given the similarity in disease etiology. Surface functionalization such as PEGylation, wherein the NP surface is covalently or non-covalently modified with a PEG pendant group, render the NPs a more neutral zeta potential and have been shown to enhance transport across the mucosal barrier and improve bronchial clearance of particles (Lai et al., 2009; Blanco et al., 2015).

Recent work has shown that nanomaterials can improve efficacy and pharmacological effect and activity of aerosolized steroids for asthma (Sahib et al., 2011; Rijt et al., 2014). Researchers developed polymeric micelles comprised of PEG(5000)-1,2-Distearoyl-sn-glycero-3-phosphoethanolamine (DSPE) encapsulated with budesonide (BUD-SSMs) via the coprecipitation and reconstitution method to study the physicochemical and pharmacodynamic characteristics of BUD-SSMs delivery for COPD (Sahib et al., 2011). BUD-SSMs demonstrated prolonged dissolution behavior compared to that of budesonide inhalation suspension (Pulmicort Respules). Furthermore, intratracheal delivery of BUD-SSMs 23 h before challenge (1 mg/kg) in an asthmatic/COPD rat model resulted in a significant decrease in inflammatory cell counts in bronchoalveolar lavage fluid compared with the exogenous delivery of Pulmicort Respules (Sahib et al., 2011). In a materials-focused study, researchers evaluated the structure-property relationship of a saturated egg phosphatidylcholine (EPC) and cholesterol (CHOL) liposome encapsulated with the anti-asthmatic drug Ketotifen fumarate (KF) delivered as a DPI (Joshi and Misra, 2001). The liposome was prepared by lipid film hydration and sonicated to have a hydrodynamic diameter of less than 5 microns and lyophilized with sucrose as a cryoprotectant (Joshi and Misra, 2001). The work demonstrated successful fabrication and delivery of the DPI liposomal KF to the necessary sites in the lung (Joshi and Misra, 2001). As previously alluded to, the size, density, and physico-chemical properties of NPs are imperative to permeate across the airways and to inflammatory cells (neutrophils) (Vij et al., 2016). Researchers assessed the delivery and efficacy of non-steroidal anti-inflammatory drug-(NSAID, ibuprofen) from PEGylated immuno-conjugated PLGA-NPs (PINP) to neutrophils (Vij et al., 2016). The size, shape, surface-properties, and targeting ability was characterized via dynamic light scatting (DLS), transmission electron microscopy (TEM), and flow cytometry (Vij et al., 2016). In short, results showed that the delivery of the drug to neutrophils in murine models of obstructive lung diseases was able to control neutrophil-inflammation and subsequent lung disease thereby highlighting the potential clinical utility of polymeric NPs for asthma and COPD (Vij et al., 2016). In a study targeted to repair the ECM, researchers utilized BSA NPs encapsulated with DOX to regenerate the degraded elastic tissue for emphysema (Parasaram et al., 2016). The NPs were optimized for their size (∼175 nm), surface properties (-60 mV), yield (∼34%), drug encapsulation efficiency (∼17%), and release properties (∼9.7% over 24 h and ∼14% over 48 h) (Parasaram et al., 2016). Release kinetics of DOX from the BSA NPs led to significant inhibition of MMPs in the lung for up to 4 weeks *in vivo* utilizing Sprague Dawley rat models (Parasaram et al., 2016). Regenerative nanotherapeutics such as the work by Parasaram et al. (2016) could serve as first-line active-based treatments thereby greatly addressing an unmet medical need for COPD treatment.

## Immunotherapy to Modulate the Lung Microenvironment: Challenges and Opportunity

**FIGURE 3:**
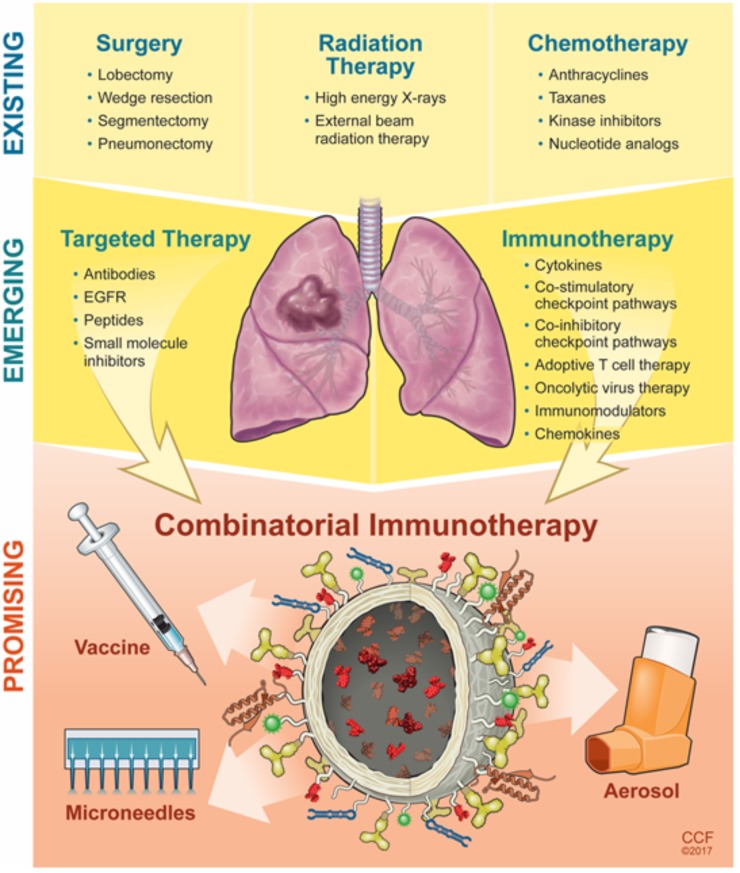
Evolution and progress of next-generation strategies to modulate the lung microenvironment.

Immuno-oncology is an emerging field that is actively being pursued by a large, diverse community who see the translational potential of targeted immunotherapeutics for cancer treatment. The investments made by pharmaceutical companies such as Novartis, AstraZeneca, Bristol-Myers Squibb, Eli-Lilly, Genentech, Merck, Pfizer, and others have pushed the immunotherapy field to new frontiers (**Table 1**). Despite its promise for active treatment against lung cancer, immunotherapies have several limitations that are not yet completely addressed such as challenges to optimal accumulation of therapeutics, dosage, and the ability to stimulate an effective immune response within the local tissue area without adverse side effects on normal tissues. The response rate to immunotherapies is still low which could be due to the complexity of the host-immune tumor interaction and presence of various tumor-mediated immune suppression mechanisms, which require further understanding. Development of predictive biomarkers and combinatorial immunotherapy approaches could alleviate such drawbacks and improve patient outcomes for those with late-stage diagnosis (**Figure 3**) (Brinker, 2012; Zang et al., 2017). NPs can provide a platform to further improve these outcomes (Shao et al., 2015). The structural compositions of and surface modifications to NPs can protect and potentiate the effects of immunotherapeutics to elicit effective responses against tumor cells (Gajewski et al., 2013). Furthermore, surface ligands presented on NPs can be targeted to specific tumor markers to facilitate local accumulation of therapeutics (Langer, 2015). Despite the emergence, development, and commercialization of a wide array of nanomaterials, bioconjugation techniques, and pharmaceutics toward treating NSCLC and/or COPD, there remain several key challenges that must be overcome, specifically from a toxicity, delivery, economical, and regulatory standpoint (Oberdörster et al., 2005; Maynard et al., 2011; Bakand et al., 2012; Barenholz, 2012; Dawidczyk et al., 2014).

Nanomaterial carriers may overcome solubility or stability issues for the drug and minimize any side effects; however, there could be toxicity issues with the material that may need to be resolved. The issue of toxicity becomes crucial for intravenously infused NPs as particle size determines their tissue distribution. Additionally, it must be ensured that the degradation byproducts of the material do not pose an adverse effect on the host-material interaction. This has brought about the field of nanotoxicology which seeks to address the interactions between nanomaterials and biological systems (Oberdörster et al., 2005). Preliminary studies from this field have led to the speculation that nanomaterials may contribute to the formation of free radicals, damage of brain cells, and undesirable penetration through the epidermis or other areas of the body. Dose escalation studies need to be performed when elucidating NP toxicity to enable successful translation.

Nanomaterial therapeutics have the potential to overcome the limitations of current standard of care therapies and offer the opportunity to reformulate discontinued drugs to deliver them in a safe and efficient manner to treat NSCLC and/or COPD. Increased efforts in basic science and translational research will greatly expedite nano-immunotherapies and initiate clinical trials toward NSCLC treatment. The National Cancer Institute (NCI) has established the cancer Nanotechnology Characterization Portal (caNanolab), which is a database that provides information on nanomaterial composition, physic-chemical characterizations, *in vitro* characterizations (cytotoxicity, blood contact properties, immune cell functions), nanomaterial pharmacokinetic properties, nanomaterial characterization, nanomaterial synthesis, publications, start-up companies, and safety reports. Bringing nanomedicine to the market presents challenges from a manufacturing, regulation, and funding standpoint. Regulatory agencies such as the Food and Drug Administration and European Medicines Agency have been collaborating on procedures for approving nanopharmaceutics. Despite these challenges, the cancer nanomedicine field is finding success due to the emergence of lab-based start-up ventures, (**Table 3**). Developing combinatorial strategies for patients suffering from NSCLC and COPD is greatly needed and can be achieved by leveraging advancements in nanomedicine, aerosol delivery strategies, and immunotherapy. With the emergence of new technology for lung resections, need for high resolution contrast agents for imaging, and the desire for localized and targeted delivery into the TME, the encapsulation of fluorophores, iron oxide agents, therapeutics or their respective combination in NPs can further aid in the translation of theranostics utilizing nanotechnology as a platform to simultaneously improve NSCLC and COPD outcomes.

**Table 3:** Sampling of start-up Companies from the NCI: Company Name and Nanomaterial Technology.

Company	Location	Nanomaterial Technology
Calando Pharmaceuticals	Pasadena, CA, United States	NP targeted delivery of siRNA and small molecule therapeutics for oncology
MagArray	Sunnyvale, CA, United States	Magneto-nanosensor technology to detect biomarkers or biomolecules labeled with SPIONS
Zymera	Palo Alto, CA, United States	Self-illuminating quantum dots for imaging, cell tracking, and tracing blood and lymphatic fluid flow
Valence Therapeutics	Evanston, IL, United States	NP delivery of chemotherapeutics for oncology
Avidimer Therapeutics	Ann Arbor, MI, United States	Therapeutic delivery from targeted dendrimers for epithelial cancer
Liquidia Technologies	Morrisville, NC, United States	PRINT technology of NPs for oncology
Xintek	Morrisville, NC, United States	CNT-based field emission technology to aid in medical imaging
MicroCHIPS Inc.	Bedford, MA, United States	Micro-chip implant for localized and sustained drug delivery
Celldex Therapeutics	Needham, MA, United States	Targeted therapeutics for immunotherapy

## Author Contributions

DS planned and wrote the manuscript. AR edited the manuscript.

## Conflict of Interest Statement

The authors declare that the research was conducted in the absence of any commercial or financial relationships that could be construed as a potential conflict of interest.
